# Robotic Peritoneal Flap Revision Vaginoplasty: Step-by-Step Technique: Tips & Tricks

**DOI:** 10.1007/s00192-026-06617-4

**Published:** 2026-04-13

**Authors:** V. Celis, V. I. Rodríguez, L. K. Fumero, L. Dubey, L. G. Medina, M. Maas, F. Eskenazi, A. Lusinchi, L. R. Doumanian, R. Travieso, R. Sotelo

**Affiliations:** 1 The Catherine and Joseph Aresty Department of Urology, USC Institute of Urology, Keck School of Medicine, University of Southern California, 1441 Eastlake Ave., Suite 7416 90089-9178, Los Angeles, CA USA; 2 Department of Urology. CIMA Hospital, University of Sonora, Hermosillo, Sonora México; 3 Division of Plastic and Reconstructive Surgery, Keck School of Medicine, University of Southern California, Los Angeles, CA USA

**Keywords:** Vaginoplasty, Peritoneal flap, Robotic surgery, Transgender, Surgical technique, Feminizing genital reconstruction

## Abstract

**Introduction and Hypothesis:**

Penile inversion vaginoplasty is the most common method utilized for feminizing genital reconstruction in transgender women. However, vaginoplasty has high revision rates. The most common functional reason for revision is stenosis or shortening of the vaginal canal, which can be challenging to perform.

**Methods:**

We present the step-by-step technique of a robotic peritoneal flap revision vaginoplasty. We discuss the indications and challenges of this surgery. Detailed steps and helpful hints are included. Finally, we outline the postoperative management and expected outcomes.

**Results:**

The robotic peritoneal flap vaginoplasty revision is feasible and safe with total operative time of 3 h, and estimated blood loss of 20 ml. The postoperative pathway includes vaginal packing and Foley catheter removal, as well as discharge within the first week. Serial vaginoscopies are later performed to assess adequate healing and patency of the new perineal pouch.

**Conclusions:**

Finding a viable harvesting site after previous perineal flap surgery is feasible. The robotic revision of peritoneal flap vaginoplasty is an appropriate procedure for re-establishing vaginal canal patency and depth.

**Supplementary Information:**

The online version contains supplementary material available at 10.1007/s00192-026-06617-4.

## Introduction

Approximately 44 million individuals worldwide are diagnosed with gender dysphoria, with gender affirmation surgery being the most effective treatment. Among various techniques, penile inversion vaginoplasty is the most common method utilized for feminizing genital reconstruction in transgender women [1, 2]. However, vaginoplasty has high revision rates, ranging from 27% to 60%, and indications varying from cosmetic to functional [3].

The most common functional reason for revision is stenosis or shortening of the vaginal canal, with a reported incidence of 12% [3, 4]. This primarily occurs owing to inconsistent or inappropriate use of vaginal dilators [4]. A typical range of 12–16 cm (5–6.5 inches) is expected, so a value lower than this [4] may necessitate revision vaginoplasty. Such revisions can be technically challenging due to the lack of available tissue and scarring from previous dissection. Additionally, limited visualization may constrain the perineal approach.

## Methods

We present the step-by-step technique of a robotic peritoneal flap revision vaginoplasty. We discuss the indications and challenges of this surgery. Detailed steps and helpful hints are included. Finally, we outline the postoperative management and expected outcomes.

## Results

The robotic peritoneal flap vaginoplasty revision is feasible and safe with total operative time of 3 hours, and estimated blood loss of 20 ml. The postoperative pathway includes vaginal packing and Foley catheter removal, as well as discharge within the first week. Serial vaginoscopies are later performed to assess adequate healing and patency of the new perineal pouch.

## Conclusions

Finding a viable harvesting site after previous perineal flap surgery is feasible. The robotic revision of peritoneal flap vaginoplasty is an appropriate procedure for re-establishing vaginal canal patency and depth.

## Supplementary Information

Below is the link to the electronic supplementary material.

Supplementary file1 (MP4 38585 KB)
